# A New Era of Muscarinic Acetylcholine Receptor Modulators in Neurological Diseases, Cancer and Drug Abuse †

**DOI:** 10.3390/ph18030369

**Published:** 2025-03-05

**Authors:** Helena Tsimpili, Grigoris Zoidis

**Affiliations:** Department of Pharmacy, Division of Pharmaceutical Chemistry, School of Health Sciences, National and Kapodistrian University of Athens, Panepistimiopolis Zografou, 15771 Athens, Greece; tsimpilieljm1@gmail.com

**Keywords:** CNS, cholinergic system, muscarinic receptors, Alzheimer’s disease, schizophrenia, drug abuse, cancer

## Abstract

The cholinergic pathways in the central nervous system (CNS) play a pivotal role in different cognitive functions of the brain, such as memory and learning. This review takes a dive into the pharmacological side of this important part of CNS function, taking into consideration muscarinic receptors and cholinesterase enzymes. Targeting a specific subtype of five primary muscarinic receptor subtypes (M1-M5) through agonism or antagonism may benefit patients; thus, there is a great pharmaceutical research interest. Inhibition of AChE and BChE, orthosteric or allosteric, or partial agonism of M1 mAChR are correlated with Alzheimer’s disease (AD) symptoms improvement. Agonism or antagonism on different muscarinic receptor subunits may lessen schizophrenia symptoms (especially positive allosteric modulation of M4 mAChR). Selective antagonism of M4 mAChR is a promising treatment for Parkinson’s disease and dystonia, and the adverse effects are limited compared to inhibition of all five mAChR. Additionally, selective M5 antagonism plays a role in drug independence behavior. M3 mAChR overexpression is associated with malignancies, and M3R antagonists seem to have a therapeutic potential in cancer, while M1R and M2R inhibition leads to reduction of neoangiogenesis. Depending on the type of cancer, agonism of mAChR may promote cancer cell proliferation (as M3R agonism does) or protection against further tumor development (M1R agonism). Thus, there is an intense need to discover new potent compounds with specific action on muscarinic receptor subtypes. Chemical structures, chemical modification of function groups aiming at action enhancement, reduction of adverse effects, and optimization of Drug Metabolism and Pharmacokinetics (DMPK) will be further discussed, as well as protein–ligand docking.

## 1. Introduction

Acetylcholine (Ach) is a neurotransmitter that is released in the brain, autonomic ganglia, and neuromuscular junctions. It affects neuronal excitability, presynaptic neurotransmitter release, and the coordination of group neuron activity. Acetylcholine controls brain activity by binding to either pre- or postsynaptic muscarinic receptors [1]. Moreover, it demonstrates multiple neuromodulatory functions in the growing and adult brain, such as regulating cortical reactions to environmental stimuli, defining arousal state, and directing neuronal development [2]. A subfamily of G protein-coupled receptors (GPCRs) known as muscarinic acetylcholine receptors control several essential central and peripheral nervous system processes. Currently, there is growing interest in studying the crystallization and pharmacology of the receptor in more depth due to the emerging need for novel therapeutic agents. Via crystallography, the different conformations provided a site to discover allosteric modulators and subtype selective agonists or antagonists, which may provide therapeutic solutions for many diseases [3]. In this review, we focus on AD, cancer, and drug abuse due to the highly unmet medical needs for the pathologies, the unique mechanistic role of muscarinic receptors, and the high potential for novel therapies. Their involvement in neurodegeneration, dopaminergic dysfunction, and oncogenic signaling presents opportunities for groundbreaking therapies that could revolutionize treatment in these fields.

## 2. The Cholinergic System

ACh is a neurotransmitter that is used by the cholinergic system to send messages and signals between the peripheral and central nervous systems (PNS and CNS). The cholinergic system is essential for many physiological processes, including mobility, cognition, memory, attention, and autonomic functions [4].

This system consists of cholinergic neurons and cholinergic receptors. Production and release of ACh is controlled by cholinergic neurons. They are classified as cholinergic neurons of the brainstem and basal forebrain. The latter, which are found in the basal forebrain area, have a significant impact on cognitive functions, learning, and memory by widely projecting to important brain regions like the cortex, hippocampus, and thalamus [5]. On the other hand, brainstem cholinergic neurons are found in particular nuclei, like the laterodorsal tegmental and pedunculopontine nuclei, expanding their control to arousal; motor coordination; and sleep–wake cycles in areas such as the midbrain, hindbrain, thalamus, and cerebellum [6].

Cholinergic receptors are proteins on cell surfaces that can be activated by acetylcholine having the role of the agonist. There are two types of cholinergic receptors: muscarinic and nicotinic receptors. Nicotinic receptors are found in both the central and peripheral nervous system, and they are ligand-gated ion channels. The activation of these receptors induces an influx of cations like sodium and calcium, resulting in excitatory responses in postsynaptic cells. In the peripheral nervous system, postsynaptic nicotinic acetylcholine receptors (nAChRs) are crucial for modulating neuromuscular and autonomic neurotransmission. In the brain, both presynaptic and postsynaptic nAChRs contribute to neurotransmission regulation and also underlie the addictive attributes associated with nicotine [7]. Muscarinic receptors, which will be this review’s primary focus, are G protein-coupled receptors and are more abundant in the CNS, but can also be found in peripheral tissues. Activation of muscarinic receptors triggers intracellular signaling cascades that modulate various cellular processes, which will be described in more detail in the following sections, with a specific focus on drugs acting on these receptors [8].

Cholinergic drugs can act either directly or indirectly. Cholinergic agonists act directly by binding to muscarinic receptors and activating them, for instance, choline esters like acetylcholine and bethanechol and alkaloids including muscarine and pilocarpine. On the other hand, there are compounds that act indirectly by inhibiting the catabolism of ACh, thus increasing its available amount. These agents can be further divided into reversible agents, e.g., physostigmine and neostigmine and irreversible agents such as echothiopate and malathion [9].

The dysfunction or degeneration of cholinergic neurons or abnormalities in cholinergic receptor signaling have been implicated in various neurological disorders. Therefore, targeting the cholinergic system with drugs that modulate cholinergic neurotransmission is an important therapeutic approach [10]. However, anticholinergic drugs may cause non-specific anticholinergic side effects, for instance, dry mouth, constipation, blurred vision, tachycardia, urine retention, sleepiness, and disorientation. Anticholinergic medicines are closely linked to cognitive impairment and are classified as potentially unsuitable medications. Compared to non-users, older people who take anticholinergic medications exhibit more pronounced deficits in memory, psychomotor speed, and cognitive flexibility. Because aging causes physiological and pathological changes, such as a decline in physiological reserve and modifications to pharmacokinetic and pharmacodynamic variables, older people are more susceptible to negative effects from medications. Age-related alterations in the body’s physiology have an impact on pharmacokinetic parameters, including decreased clearance of various medications, an increase in the volume of distribution for fat-soluble drugs, and body composition. These modifications raise the aged population’s chance of experiencing negative reactions to routinely used drugs. Additionally, older persons have decreased hepatic metabolism and renal excretion of medicines, which may lead to greater anticholinergic effects and drug accumulation in tissues. Furthermore, permeability of the blood–brain barrier (BBB) has to be considered, since it increases with age. Additionally, aging is linked to increased pharmacodynamic sensitivity to CNS muscarinic receptor blockade due to a decrease in cholinergic reserve in the aging brain and structural changes in the muscarinic receptor binding sites that affect the acetylcholine binding affinity. Aging may also negatively affect the expression of muscarinic receptor subtypes or the plasticity of muscarinic receptor-mediated signal transduction [11].

## 3. The Muscarinic Receptor

Muscarinic receptors are present in two main locations within the peripheral parasympathetic neurons. Firstly, they are found presynaptically at the nerve endings, preventing the release of neurotransmitters. Secondly, these receptors are present in autonomic ganglia, where they contribute as an additional inhibitory element in transmission and generate a gradual depolarization of the ganglionic cell body. Muscarinic receptors are broadly dispersed throughout the brain in addition to these sites. Pharmaceutical substances have the ability to interact with muscarinic receptors in two individual ways: as agonists, imitating the effects of naturally occurring ACh, or as antagonists, obstructing regular parasympathetic functions and promoting the dominance of sympathetic activity [12]. The broad distribution of muscarinic receptors across various locations and their distinct responses to stimulation or inhibition have led to the development of diverse therapeutic applications. They moderate the physiological effects of parasympathetic nerve activity and are mostly found in post-synaptic cell membranes of smooth muscle, cardiac muscle, and glandular tissue. The effects of activating muscarinic receptors can be inhibited by atropine, a muscarinic antagonist [13]. There are intense research efforts aimed at discovering new potent and selective compounds, with an ultimate goal of minimizing the adverse effects. The common skeletal structure of compounds having muscarinic activity is the quaternary ammonium salt and the heteroatom existing within distance of 4.4 Å. The interest of research is focused on molecules with higher efficacy and selectivity and fewer side effects [14]. There are five primary subtypes of muscarinic receptors: M1, M2, M3, M4, and M5. Although every subtype is present in the central nervous system, they are found in different tissue types and are encoded by different genes. The stomach, salivary glands, and cerebral brain are the main locations for M1 receptors. M2 receptors are present in heart tissue and smooth muscles. Salivary, stomach, and smooth muscle glands also have M3 receptors. M4 and M5 receptors are found in the substantia nigra and hippocampal regions; however, they are less characterized [15]. Thus, efforts to selectively target different subtypes arouse great pharmaceutical research interest [16].

The high degree of similarity among the five mAChR subtypes has hindered the drug development of selective and effective ligands. All subtypes share a large number of conserved residues, especially in the seven transmembrane helices. Remarkably, a different residue in the M2 and M4 subtypes and the same residue in the M1, M3, and M5 subtypes occupy some of the same places. On the other hand, aside from a brief Ser/Thr-rich region, the vast third cytoplasmic loop exhibits almost no similarity among the mAChR subtypes. However, it is well known that even slight variations in a single GPCR sequence can result in notable variations in binding site design and, consequently, ligand affinities [17].

**Figure 1:**
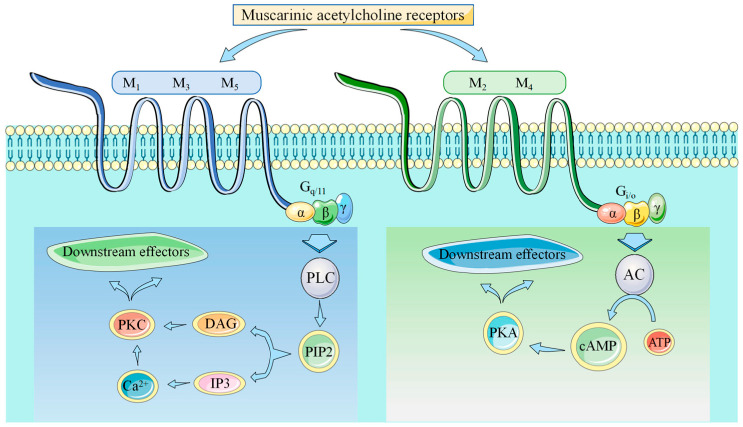
Muscarinic receptors signaling pathways, from [18].

**Figure 2:**
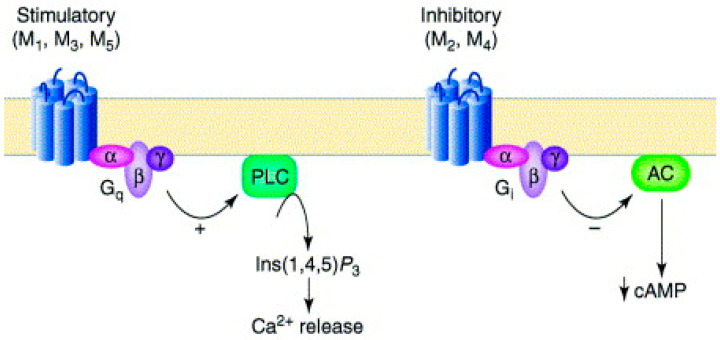
Muscarinic receptors’ stimulatory and inhibitory actions, from [19].

GPCRs comprise all muscarinic receptors. Depending on the type of G protein that is present in the receptor, ligand binding following GPCR activation results in the production of second messengers. Excitatory Gq receptors produce protein kinase C (PKC) and phospholipase C. Diacylglycerol (DAG) and inositol triphosphate (IP3), the second messengers, are subsequently produced by phospholipase C. DAG and IP3 function to raise intracellular calcium and protein kinase, which supply the excitatory reaction’s mechanism. Gi receptors are inhibitory, which means that they lower adenylyl cyclase, which in turn lowers protein kinase A. The reduction in protein kinase A leads to a decrease in cyclic adenosine monophosphate levels within the cell, which triggers an inhibitory response (Figure 1). M2 and M4 receptors are inhibitory receptors consisting of the Gi protein, whereas M1, M3, and M5 receptors are stimulatory receptors consisting of the Gq protein (Figure 2) [15].

**Figure 3:**
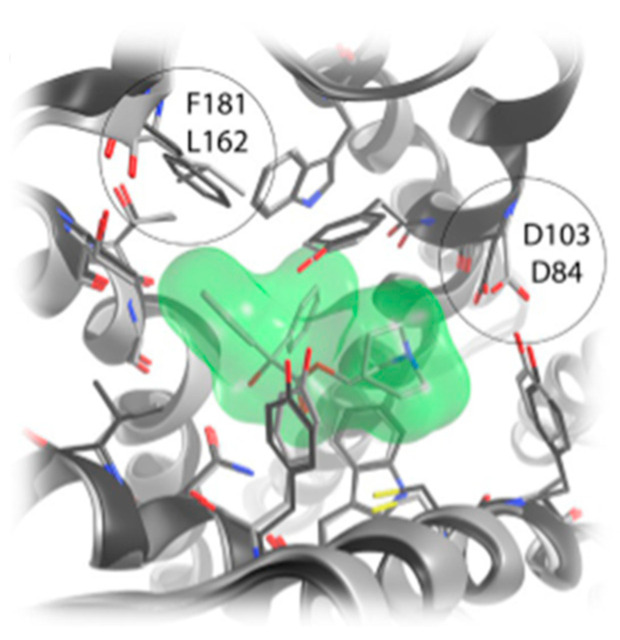
Difference in residue existing in EL2 between M2 and the other subtypes, from [19].

G-proteins are comprised of three subunits: α-, β-, and γ-. Based on the α subunit’s major sequence homology, the G protein is categorized as Gs, Gi/o, Gq, or G12. The inhibitory mechanism of M2 and M4 receptors is caused by decreasing adenylate cyclase activity and delaying the opening of potassium, non-selective cation, and transient receptor potential channels. Muscarinic M1, M3, and M5 receptors mobilize phosphoinositides to produce inositol 1,4,5-trisphosphate and 1,2-diacylglycerol, leading to a rise in intracellular calcium [20]. The M2 receptor is the first structurally described cholinergic receptor in humans. About two-thirds of the way down the membrane, a lengthy aqueous channel is where the antagonist 3-quinuclidinyl-benzilate attaches. In all five muscarinic receptor subtypes, the amino acids that make up the orthosteric binding pocket are the same. Tyrosine residues accumulate to create an aromatic cap that prevents the attached ligand from dissociating. Residues at the binding pocket’s entrance close to this aromatic cap have been identified as an allosteric ligand binding site [21]. The orthosteric sites of all muscarinic receptors show a high conservation; hence, the need for discovering selective drugs targeting a specific subtype is crucial. The extracellular side of the mAChRs has an allosteric vestibule that varies depending on the subtype. Using allosteric or dualsteric modulators to target this location is a good way to achieve subtype selectivity. For every subtype, there is a strong resemblance between the highly conserved transmembrane sections and the orthosteric binding pocket, but M2 receptors have a significant difference. F181, a distinct residue near the end of extracellular loop EL2, aids in orthosteric ligand binding. This is where leucine is in all other subtypes (Figure 3).

**Figure 4:**
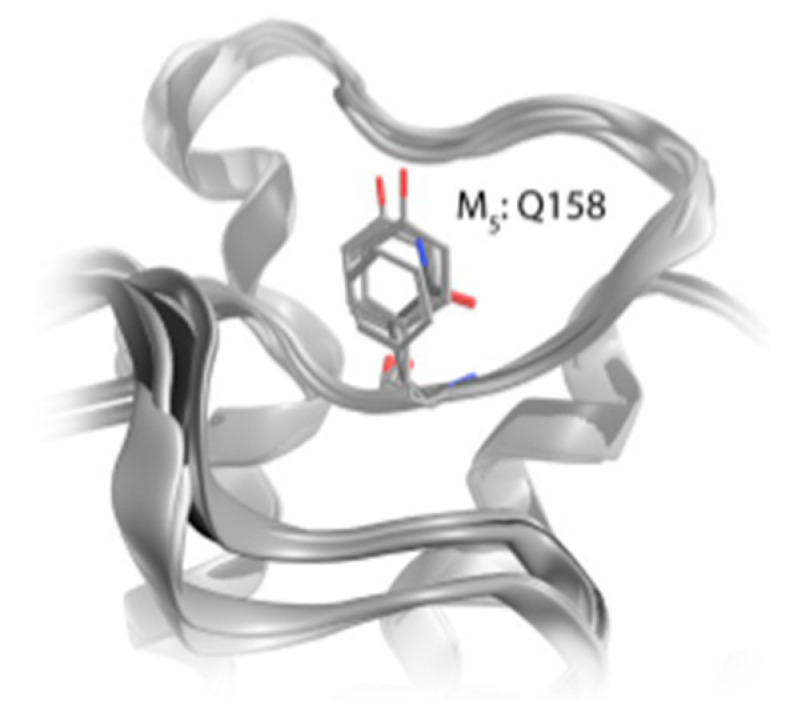
Presence of glutamine in the middle of EL2 in M5 receptor, from [22].

In addition, three nearby acidic residues may be seen in the M2 receptor’s EL2 region. Among the five subtypes of the EL2, this segment is one of the most variable ones. In the middle of EL2, the M5 receptor has a different characteristic due to the presence of glutamine instead of a typical aromatic residue (Figure 4) [22]. It is clear that modulation of the allosteric binding site outlines great significance for drug discovery.

## 4. Acetylcholine as a Neuromodulator

**Figure 5:**
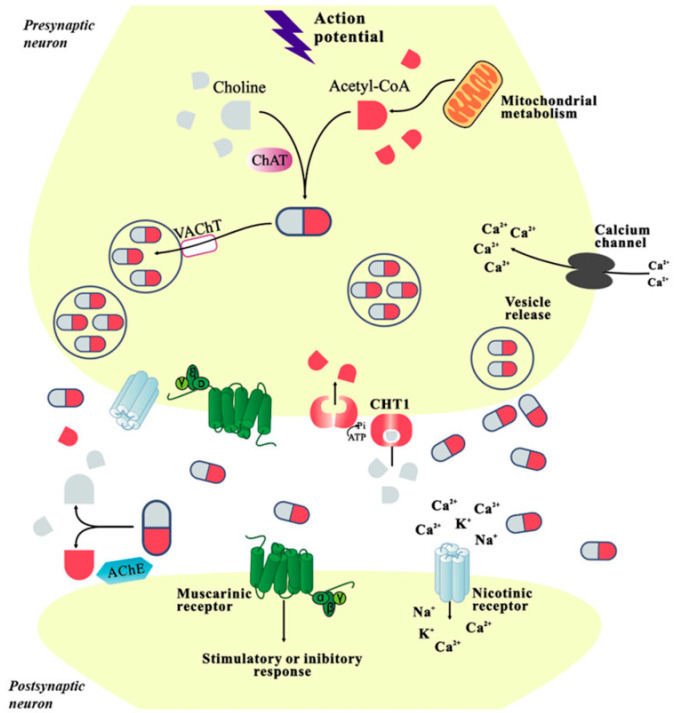
Acetylcholine neurotransmission, from [25].

Acetylcholine (ACh) is a neurotransmitter that acts quickly and point-to-point in the autonomic ganglia and neuromuscular junction. Despite being the main excitatory neurotransmitter in the peripheral nervous system, ACh seems to function as a neuromodulator in the brain [13]. Normal cortical sensitivity to external stimuli is increased by ACh, which also reduces corticocortical transmission and increases focused attention. However, elevated ACh signaling can cause symptoms associated with anxiety and sadness. For instance, prolonged elevations in cholinergic transmission may result in maladaptive behaviors, whereas stress-induced ACh release can lead to adaptive responses to environmental stimuli [23]. Synthesis of ACh is catalyzed by choline acetyltransferase (ChAT), which is responsible for transferring the acetyl group from acetyl-CoA to choline. The vesicular acetylcholine transporter (VAChT) contributes to the effective storage of ACh by facilitating its encapsulation into synaptic vesicles. After depolarization of the nerve, ACh is exocytosed and travels across the synaptic cleft to interact with postsynaptic receptors (Figure 5) [24].

ACh additionally plays a role in modulating inflammation, with evidence indicating that ACh sourced from parasympathetic innervation can hinder inflammatory cytokines release from macrophages through nicotinic receptor activation, suggesting a “cholinergic anti-inflammatory pathway” [26]. Many neurodegenerative diseases, including Alzheimer’s, Parkinson’s, and Huntington’s, as well as psychiatric disorders like schizophrenia, have been linked to altered levels of ACh or altered receptor expression and function in specific nervous system locations. The cognitive, behavioral, and motor impairments that are typical of these diseases are often linked to cholinergic circuit dysfunction [27].

## 5. Targeting the mAChRs: Alzheimer’s Disease

Alzheimer’s disease (AD) is a neurodegenerative condition marked by tau-containing intracellular neurofibrillary tangles and extracellular plaques that contain β-amyloid (Aβ). Although amnestic cognitive impairment is the most common presentation of AD, non-amnestic cognitive impairment is also a symptom, though less common. The serious results caused by cognitive dysfunction vary. Early symptoms consist of weakness in mental performance tests. Mild cognitive impairment (MCI) is characterized by one less effective mental domain or by multiple ones, whereas there is no impairment in the effective/functional ones [28].

An accumulation of aberrant neuritic plaques and neurofibrillary tangles in the brain is a pathological hallmark of Alzheimer’s disease. Neurons, especially cholinergic neurons in the neocortex and basal forebrain, are lost in conjunction with these pathogenic alterations. There are two main pathophysiological suggestions: the Cholinergic suggestion and the Amyloid one. The cholinergic hypothesis is explained by the reduction in neurons and the subsequent reduction in ACh levels. This proposal underlines the important role of ACh in cognitive function. Beta-amyloid seems to disrupt the cholinergic function by impairing the release of ACh and thus promoting cholinergic synaptic loss. The Amyloid proposal is believed to be the pathophysiological reason for Alzheimer’s Disease (AD). According to the amyloid hypothesis, β- and γ-secretase enzymes work to convert amyloid precursor protein (APP) into amyloid beta (Aβ) peptide. Alpha- or beta-secretase may often cleave APP, and the resulting little fragments are not harmful to neurons. However, if beta- and gamma-secretase cleave APP sequentially, 42 amino acid peptides (Aβ42) are produced. Neuronal toxicity results from amyloid aggregation caused by elevated Aβ42 levels. Instead of normal APP breakdown, Aβ42 promotes the development of aggregated fibrillary amyloid protein [29].

**Figure 6:**
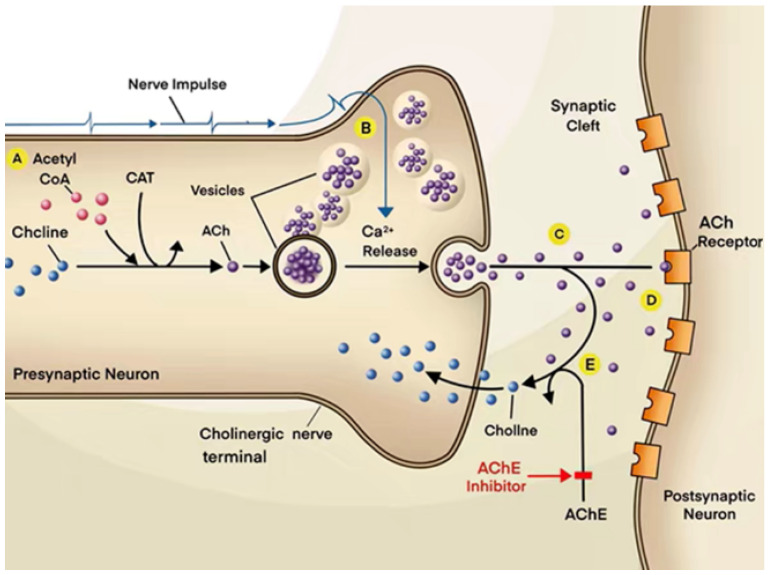
Mechanism of action of AChEIs. (A). Acetylcholine synthesis in nerve terminals from acetyl coenzyme A (acetyl CoA) and choline, in a reaction catalyzed by choline acetyltransferase (CAT). (B). Ca^+2^ get into the cell during a synapse. (C). ACh is released from the vesicles and into the synaptic cleft. (D). ACh binds to receptors on the postsynaptic neuron. (E). AChE concurrently breaks down ACh. As a result, less information is transmitted since less ACh binds to receptors. Today, AChE inhibitors are utilized as medicine to address this problem, from [25].

There are many potential therapies for AD (Table 1). Among them, acetylcholinesterase and butyrylcholinesterase are a target for AD treatment. Inhibition of AChE and BChE can prolong the circulation of ACh and thus the cholinergic signaling (Figure 6). The only clinical medications currently authorized for the treatment of AD patients are AChEIs. Initially, tacrine was administered as a first potential therapy solution, but the administration was disrupted. The most used AChEIs are the second-generation medicines donepezil, galantamine, and rivastigmine [25].

Donepezil may raise the hippocampal protein levels of PTEN-Induced Kinase 1 (PINK1), Neurofascin (NFASC), Myosin Light-Chain Kinase 2 (MYLK2), and Neuroblastoma RAS viral oncogene homolog (NRAS). There are several ways that donepezil can function, and each one is crucial for protecting the nervous system and neurons. PINK 1 is specifically linked to cellular protective mitochondrial failure and mitochondrial autophagy, both of which may play a major role in the pathophysiology of AD. Another therapy may come from alkaloids of plants, such as galantamine, which may enhance the cholinergic system activity. Beta-secretase (also known as BACE1) and Y-secretase endoproteolyze their APP to create β-Amyloid (Aβ). Alternative APP cleavage by α-secretase (a membrane-bound family of metalloproteinases) results in the creation of neurotrophic and neuroprotective secretory sAPPa fragments and stops the synthesis of harmful Aβ. Rivastigmine directs APP therapy away from BACE1 and toward α-secretase, in addition to its anticholinesterase action [25].

Particularly noteworthy is the role of PINK1, which has been associated with mitochondrial autophagy and the mitigation of cellular damage resulting from mitochondrial dysfunction. This finding holds significant relevance in the context of AD pathogenesis, as mitochondrial dysfunction is known to be a contributing factor in the disease. The diverse mechanisms of action exhibited by donepezil, including the upregulation of crucial proteins like PINK1, emphasize its potential as a valuable therapeutic agent for preserving neuronal integrity and combating the underlying neurodegenerative processes in AD [52].

Tacrine is subject to first-pass metabolism in the liver and extensively processed by the cytochrome P450 system, believed to be the cause of its elevated hepatotoxicity. It functions by impeding the breakdown of acetylcholine, leading to its extended activity and increased levels in the cerebral cortex [53]. Despite 9-amine-1,2,3,4-tetrahydroacridine (THA) being highly hepatotoxic, its flexibility to chemical modification has rendered it a widely employed framework for more recent drug development [54]. In the process of designing multitarget-directed tacrine derivatives, the amino group can be altered by attaching other functional fragments, leading to a significant reduction in tacrine’s hepatotoxicity. Moreover, recent research has shown that homo- and hetero-dimers of tacrine can enhance its biological characteristics and even alleviate certain adverse effects [55].

Rivastigmine, on the other hand, works by inhibiting AChE, while also inhibiting butyrylcholinesterase [56]. AChE is the main cholinesterase present at nerve synaptic junctions and regions of high activity within the adult human cerebral cortex, while BChE is predominantly found in glial cells and plays a significant role in cholinergic modulation. In Alzheimer’s disease, while AChE activity decreases in the hippocampus and temporal cortex, the action of BChE increases in these regions, highlighting the significant role of BChE in regulating brain acetylcholine levels [57].

Galantamine was believed to positively allosterically modulate nicotinic receptors, in addition to its effects on the acetylcholine levels, but it has been concluded that Galantamine does not function as a positive allosteric modulator for α7 or α4β2 receptors, which are the predominant types of nACh receptors found in the mammalian brain [58]. Although there have been some clinical trials comparing the efficacy of these AChE inhibitors, there is no consensus on their differentiation. However, only 25–50% of patients respond to AChE inhibitor therapy [56]. The clinical efficacy of these inhibitors is limited, which may be attributed to the dysregulation of cholinergic receptors due to amyloid beta pathology. This leads to the presumption that the cholinergic dysfunction is not solely due to the loss of acetylcholine. Therefore, while acetylcholine abundance is undoubtedly significant, functional impairment of the acetylcholine receptor in the synapse may also contribute to Alzheimer’s disease [59].

Research has been conducted on agonists acting on postsynaptic mAChR within the cerebral cortex, including arecoline, RS-86, and pilocarpine, in order to improve mental activity and cholinergic signaling. Orthosteric agonists can cause endocytosis of receptors or downregulation, whereas AC-42 (Table 1) (an allosteric M1 mAChR agonist) does not affect the cell-surface expression of receptors [30,31].

These agonists still did not achieve favorable outcomes in clinical trials, so the new attempt was to enhance the clinical effectiveness of arecoline, within the cortex in comparison to ACh, by exchanging its ester group with a 3-methyl-1,2,4-oxadiazole bioisostere, demonstrating more metabolic stability (Table 1). Although the resulting arecoline-based oxadiazoles showcased higher effectiveness, they offered minimal efficacy improvements. Drug development then moved into the quinuclidine and azanorbornane-methyl and amino oxadiazoles (Table 1), which displayed significant enhancements in potency and effectiveness compared to the arecoline series. Among these, the amino oxadiazole demonstrated the greatest potency and anticipated efficacy [60]. These initial endeavors resulted in the development of a collection of mAChR agonists, which enabled the recognition and analysis of structure–activity relationships within orthosteric ligands [32].

Further studies showed that expanding the conformational flexibility and size of the azacyclic ring resulted in decreased affinity when compared to the comparatively rigid arecoline, azanorbornane, and quinuclidine cores. The surface binding site and the flexibility in conformation can affect the efficiency and binding interaction of azabicyclic ligands. Exo-1-azanorbonane seemed to have the best conformation possible, and it is one of the most efficient and highly active muscarinic agonists. The geometry between the hydrogen bond acceptor pharmacophore and steric bulk around the base and the cationic head group affects the muscarinic agonists’ efficacy and affinity in a variety of isoquinuclidine-based compounds [60]. Initially, the metabolically stable 3-methyl-1,2,4-oxadiazole bioisostere was used to replace the ester group in arecoline, a weak partial agonist in the cortex when compared to ACh, in an attempt to improve its clinical profile. The development of quinuclidine (1-azabicyclo[2.2.2]octane) and azanorbornane (1-azabicyclo[2.2.1]heptane)-methyl and amino oxadiazoles resulted from the development of arecoline-based oxadiazoles, which showed higher potency than arecoline itself but provided only slight efficacy increases [32].

The 1,2,4-oxadiazole ring was attempted to be replaced by tetrazole/1,2,3-triazole scaffolds, oxime ether functionality, 4-thiazolidinone, and ether connections directly to functionalized pyrazine rings (Table 1). All these failed in clinical trials [32].

With neurochemical analysis of brain tissue obtained from individuals with AD showing a reduction in the activity of the presynaptic marker enzyme and M2-mAChRs in various brain regions, drug development efforts shifted to the generation of functionally selective M1 agonists. The capacity of M1 receptors in frontal cortical samples of individuals with Alzheimer’s disease to create high-affinity agonist-receptor-G protein complexes that are guanine nucleotide-sensitive was lost [61].

### 5.1. M1 Muscarinic Agonists for AD

Research conducted in the early 2000s revealed that M1 muscarinic receptors are a target for the treatment of Alzheimer’s disease. Some M1-selective muscarinic agonists, such as AF102B, AF150(S), and AF267B (Table 1), are prospective drugs that can improve cognition and hinder the illness’ progression. During preclinical investigations with AF102B, M1R agonism leaded to suppression of Beta-Amyloid (Aβ) secretion from cultured cells. AD is characterized by the accumulation of Aβ deposits in the brain’s diffuse and compact senile plaques. Drug therapy for AD aims to prevent Aβ deposition, as elevated levels of Aβ could potentially harm neurons due to their toxic effects [33,62]. Activation of M1R can induce αAPPs synthesis, which prevents Aβ peptide formation. M1 agonists may inhibit the synthesis of Aβ via enhancing the “α-secretase” processing pathway in Alzheimer’s patients. Research has demonstrated that muscarinic agonists can raise αAPP secretion in vitro, which lowers the generation of Aβ. Notably, M1-selective agonists could potentially influence APP processing in brain regions like the cortex and hippocampus, where M1 receptors are abundantly present [56]. Earlier research demonstrated that AFI50(S) can bring the levels of brain ChAT and AChE in apoE-deficient mice back to the levels observed in control mice, which is traced back to the restoration of effectiveness with cholinergic synaptic activity in the brain by M1R activation [63].

It has been demonstrated that AF267B lowers the amount of amyloid in rabbits and lessens cognitive impairments, as well as lowers the tau and amyloid levels in triple-transgenic AD mice, and targets major AD hallmarks by activating protein kinase C and a disintegrin and metalloprotease 17 (ADAM17) via M1 mAChR. It was the first low-molecular-weight therapy reported to have these effects [64].

Developing drugs that target specific receptor subtypes while also having good drug-like properties and being effective, safe, and well-tolerated has been a significant challenge. Additionally, a lack of biomarkers for measuring target engagement has made it difficult to interpret clinical trials in which no important effectiveness was found, resulting in inadequate testing of the muscarinic M1 receptor hypothesis [65]. The critical function of M1-mAChR in cognitive abilities highlights the importance of developing ligands that can stimulate the receptor and alleviate cognitive impairments observed in AD [66]. A new generation of M1 receptor orthosteric agonists and positive allosteric modulators are now making their way into clinical practice, propelled by developments in structure-based drug design and an understanding of the ideal pharmacological characteristics required to provide clinical efficacy while minimizing side effects [67].

Orthosteric agonists, which bind to the same binding site as endogenous ligand ACh, offer a compelling therapeutic approach. Unlike AChEIs, the degeneration of presynaptic cholinergic neurons does not impact the function of orthosteric agonists in AD, making them potentially more effective. Partial agonists, which have low intrinsic activity, may be preferred over full agonists due to their capability to decrease agonist-induced desensitization and undesired cholinergic effects at concentrations relevant to clinical use. Several M1-mAChR orthosteric agonists, including AF267B, WAY-132983, CDD-0102A, SPP1, and xanomeline, demonstrated cognition-enhancing effects in preclinical models. Cholinergic side effects have restricted the use of xanomeline, which preferentially binds to M1 and M4 muscarinic receptors subtypes, in AD patients. However, it has shown promising results in the treatment of schizophrenia, which will be evaluated in the following sections. Recent developments in M1-mAChR orthosteric partial agonists, such as HTL9936; ethyl (4S)-4-[4-[(1-methylcyclobutyl)carbamoyl] piperidin-1-yl]azepane-1-carboxylate, have exhibited notable cognition-enhancing effects in preclinical animal models and elderly human participants, while maintaining a minimal occurrence of cholinergic adverse effects [66].

**Figure 7:**
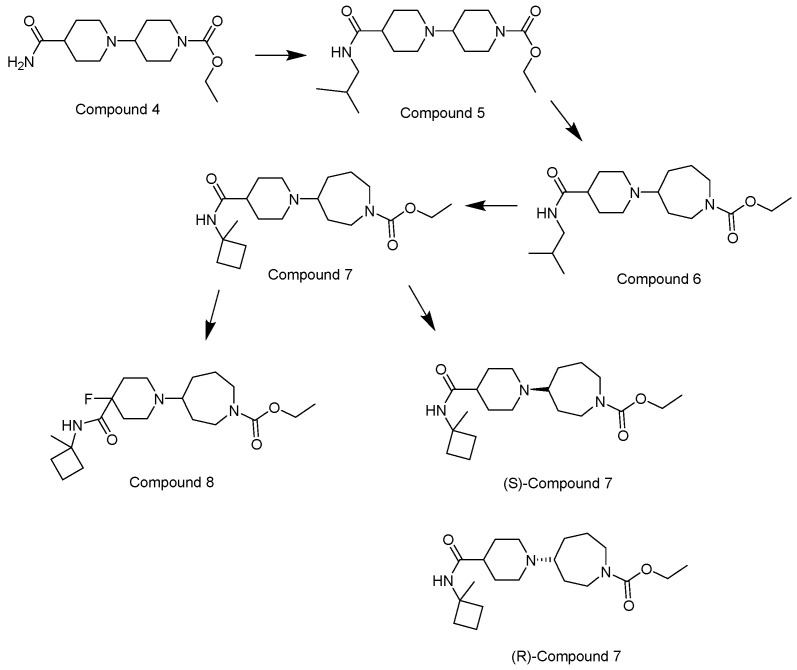
Structural modifications of the initial hit molecule Compound 4 resulted in the invention of (S)-Compound 7 (HTL9936), from [34].

Recent research and screening efforts were made, and in the methodical and progressive translational approach, the pharmacology of the selective M1 receptor orthosteric partial agonist (HTL9936) (Table 1) was cautiously profiled using Structure-Based Drug Design (SBDD) (Figure 7). The goal was to verify whether this molecule exhibited the desired characteristics without the dose-dependent adverse effects linked to prior M1 receptor agonists [34].

**Figure 8:**
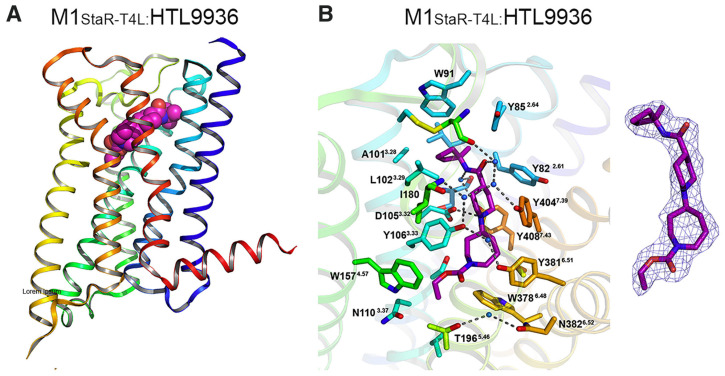
(**A**) Crystallization of interaction between M1-StaR-T4L and HTL9936, from [66]. (**B**) Binding location of M1-StaR-T4L, interaction with ligand HTL9936, from [34].

The team centered on the advancement of selective M1 receptor agonists through the synthesis of a range of small amides. The specific compound demonstrated enhanced activity as an M1 receptor agonist but remained having low activity at other receptor subtypes. The input of an azepine ring in another racemic compound resulted in sub-micromolar potency at M1R with no detectable affinity with other subtypes. Preferred secondary amides were identified, including cyclobutylmethyl secondary amide in another racemic compound and on the azepine ring; the (S)-enantiomer (HTL9936) was the favored chiral center. The M2 receptor agonist activity for GSK1034702 was found to be considerable in structural investigations of M1-StaR-T4L with HTL9936 and the M1 agonist GSK1034702, which also demonstrated that HTL9936 is predominantly retained in place by a charge–charge interaction and rotates the tyrosine cage (Figure 8). It is important to conclude that HTL9936 exhibited notable cognitive benefits in various animal models used in preclinical studies. Moreover, in older patients, HTL9936 induced minimal cholinergic adverse reactions and stimulated the areas related to memory and learning [34].

Another M1 over M4 selective orthosteric partial agonist studied recently is HTL0018318, showing positive effects in attention and memory measures. In addition to therapeutic doses of donepezil, HTL0018318 was well tolerated in individuals with mild-to-moderate Alzheimer’s disease, and it was beneficial to attention and episodic memory [68]. The co-administration with donepezil was indicated to be safe, with no pharmacodynamic or pharmacokinetic interferences of both drugs [69]. Regarding adverse effects, HTL0018318 exhibited overall good tolerability and was linked to mild-to-moderate cholinergic adverse effects. Subjects receiving HTL0018318 had marginally higher blood pressure and pulse rates than those getting a placebo, with the blood pressure increase tending to decrease with subsequent treatment [70]. Despite the encouraging data, future research needs to clarify the potential activity of HTL0018318 in the upcoming treatment of AD [69].

### 5.2. M1 Muscarinic Allosteric Ligands for AD

There has been great effort to synthesize selective agonists for mAChR because of the high conservation of the ACh binding site. This conservation makes it difficult to produce compounds that are genuinely specific to a single subtype. An alternative strategy was focused on producing molecules that target less conserved sites and a spatially different binding site with different functions. This strategy has been shown to be quite effective in recent years for creating subtype-selective ligands for several GPCRs [71].

Although early discoveries have generated curiosity in developing muscarinic ligands, there has been a shift in attention towards identifying ligands that exhibit enhanced selectivity for M1 receptors to provide safer and more effective treatment possibilities for CNS disorders. To achieve intrinsic selectivity, one promising strategy is to focus on allosteric binding sites, which are different from the cholinergic binding site. This has led to the progress of direct-acting allosteric agonists and Positive Allosteric Modulators (PAMs) [72].

Using an M1 PAM may have the benefit of changing the native receptor ligand while preserving the temporal and spatial elements of receptor signaling; in contrast, a direct-acting agonist does not depend on endogenous acetylcholine, which decreases with the progression of Alzheimer’s disease [73]. As a result, the focus of drug discovery has been on developing M1 PAMs that selectively increase acetylcholine’s action at the M1 receptor while having no significant effects on other receptor subtypes. The potential of the first-generation M1 PAM, benzyl quinolone carboxylic acid (BQCA), and the succeeding second- and third-generation M1 PAMs to improve ACh binding and function at M1 receptors without impacting M2-M3 receptors was shown by radioligand binding studies [74]. Additionally, in a transgenic mouse model of AD, BQCA corrected reversal learning deficits. Additionally, it was found to regulate non-amyloidogenic APP processing in laboratory experiments, suggesting that M1 PAMs may be able to help Alzheimer’s patients with both symptom relief and possible disease-modifying benefits [75].

This highlights the significant selectivity of M1 PAMs compared to orthosteric agonists, with BQCA being an exemplary framework for creating remarkably selective M1 mAChR PAMs [76].

However, recent crystallographic studies have unveiled that the allosteric site on the M1 receptor, which is typical of all receptors in the family, exhibits a remarkable similarity in shape and the residues lining the pocket. This raises the question of how M1 PAMs achieve their subtype selectivity despite the conservation of the allosteric pocket. A contemporary perspective suggests that most allosteric modulators, including M1 PAMs, bind to the conventional allosteric site found in all receptor subtypes. Their specificity is achieved by conveying positive allosteric effects or cooperativity to the concurrently bound orthosteric ligand, but with varying degrees varying with the specific receptor subtype [77]. This notion, known as “cooperativity-driven selectivity”, indicated that while presently recognized M1 PAMs amplify the effects of ACh specifically at the M1 receptor, they exhibit binding to the allosteric site of the majority, if not all, other receptors. However, at these other receptors, they exhibit neutral modulation effects or neutral cooperative interaction with ACh [74].

According to very recent studies, M1 PAMs like TAK-071 (Table 1) that have low cooperative interaction (α-value) are more tolerable in the gastrointestinal system than those that have greater cooperativity values. Targeting the M1 receptor, which is mostly expressed in brain regions linked to cognitive function, is TAK-071, a new M1R PAM [35]. It is anticipated that selective potentiation of this receptor’s activation will ameliorate cognitive impairments. According to research conducted on animals, TAK-071 improved the cognitive deficits caused by scopolamine and inhibited the enhancements it caused in the alpha, theta, and delta power bands of quantitative electroencephalography (qEEG) in cynomolgus monkeys [78].

Another promising study featuring PAMs offers a recently formulated highly potent M1 mAChR PAM, VU0486846, based on a novel benzomorpholine core, which has exceptional oral bioavailability and CNS penetration, and lacks direct agonist action or cholinergic toxicity [79]. VU0486846 (Table 1) exhibits weaker agonist efficacy in cell lines expressing M1 receptors with abundant receptor reserve, and it lacks agonistic effects in the prefrontal cortex (PFC). However, VU0486846 shows notable efficacy in improving cognitive function in the novel object recognition model, in contrast to agonist PAMs [36].

**Figure 9:**
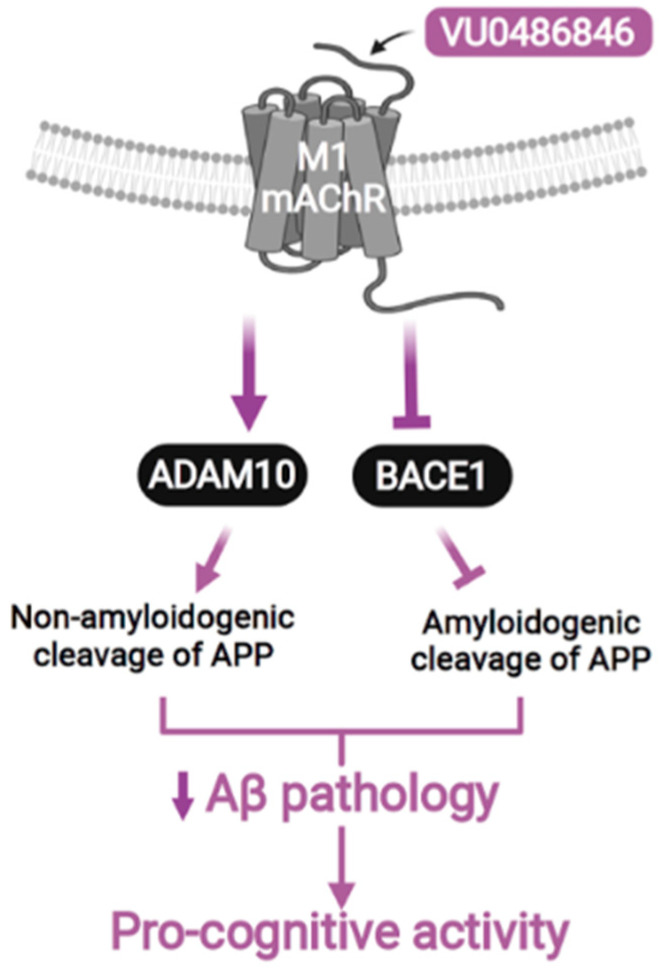
By blocking amyloidogenic cleavage via β-secretase1 (BACE1), activation of M1 mAChR with VU0486846 increases the non-amyloidogenic cleavage of amyloid precursor protein (APP) via ADAM10. This results in a decrease in the pathology caused by β-amyloid (Aβ) and supports the pro-cognitive function of VU0486846 in female AD mice, from [80].

**Figure 10:**
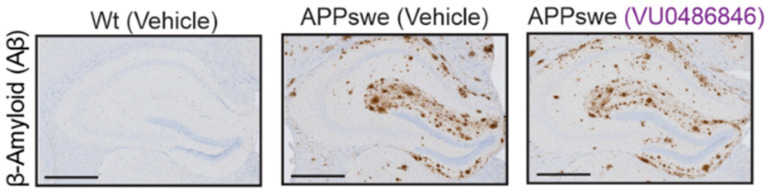
In female APPswe mice, VU0486846 improves non-amyloidogenic processing of APP and reduces Aβ pathology. Images are representative of five independent experiments (scale bar, 500 μm), from [80].

The findings show that administering VU0486846 to female APPswe mice with AD symptoms on a long-term basis improves their working memory and spatial impairments, as well as their anxiety-like behavioral patterns. Furthermore, administration of VU0486846 results in a reduction of Aβ oligomer levels in the hippocampus and a change in APP processing from amyloidogenic cleavage to non-amyloidogenic cleavage (Figure 9 and Figure 10). Consequently, as female individuals make up a larger percentage of identified cases, using novel M1 mAChR PAMs may provide a promising treatment approach to safely minimize AD progression. Nevertheless, additional research is necessary to ascertain whether this new M1 mAChR PAM maintains its effectiveness in male AD mice [80].

## 6. Targeting the mAChRs: Schizophrenia

The complex and varied behavioral and cognitive illness known as schizophrenia is thought to result from abnormalities in the brain’s development caused by genetics, the environment, or both. While dysfunction in dopaminergic neurotransmission contributes to the emergence of psychotic symptoms, indications also propose the involvement of other brain regions and circuits to varying degrees [81]. It is a serious mental illness marked by abnormalities in perception, thought, emotion, and behavior [82]. While the causes of cognitive impairment in schizophrenia are complex, central cholinergic dysfunction has been repeatedly linked to the disorder’s molecular and neural circuit abnormalities [83].

In schizophrenia patients with no medication administered, a study pinpointed the reduction in muscarinic receptors [84]. The drugs used in schizophrenia possess strong anticholinergic properties, thus leading to deterioration of cognitive function and high risk of dementia development [83]. Treating schizophrenia through targeting muscarinic receptors needs to be further researched and is a promising approach. Currently, pharmacotherapy for schizophrenia primarily relies on dopaminergic and serotonergic antagonists/partial agonists [85]. Other novel approaches in the treatment of schizophrenia, apart from the muscarinic receptor, involve exploring alternative antipsychotic treatments and identifying potential diagnostic and theragnostic biomarkers. These methods consider several theories, including neuroinflammation, the cannabinoid system, the gut–brain axis, neuromediators (dopamine, glutamate, and serotonin), and oxidative stress [86].

Both preclinical and clinical investigations indicate that directing attention towards subtypes of muscarinic acetylcholine receptors may offer a more holistic approach to alleviating symptoms, potentially improving various domains of symptoms. In-depth studies examining these mechanisms have unveiled that receptor subtypes M1, M4, and M5 possess the ability to influence the specific brain circuits and physiological processes that show disruption in schizophrenia. It is thought that these subtypes play a role in how positive, negative, and cognitive symptoms present [87]. In 1999, research revealed the properties of Clozapine (Table 1), which acts as a partial agonist but also shows activity as an mAChR antagonist on different subunits of the muscarinic receptor [37].

Irrespective of whether clozapine acts as a true antagonist or a weak partial agonist of mAChRs, its overall impact on physiological tissue is a reduction in acetylcholine (ACh) signaling through mAChRs. Multiple lines of evidence suggest that the activation of M1 receptors plays a crucial role in clozapine’s ability to modulate schizophrenia-related connections and facilitate behavioral efficacy in animal testing [88]. For patients with treatment-resistant schizophrenia (TRS), clozapine seems to be the best solution regarding efficacy and effectiveness [89]. It is proved that administration of dopamine D2 receptor blockers does not alleviate the disease, while TRS is characterized by individual neurobiology. TRS is a subsequent effect to schizophrenia, as it affects 20–50% of schizophrenia patients. Glutamate is abnormally regulated in TRS patients, and the fact that D2 blockers do not have any effect is caused by unchanged dopamine function. Clozapine seems to regulate the glutamate levels, being a significant first-line treatment for TRS. Furthermore, it acts on muscarinic, dopamine, and serotonin receptors, and the lack of binding affinity to D2 receptors is responsible for no extrapyramidal symptoms. Clozapine seems to reduce the chance of hospital readmission and the mortality rates [90].

Despite having acceptable serum levels, between 40% and 70% of individuals with treatment-resistant schizophrenia do not respond to clozapine. Many therapeutic approaches have been developed for these patients, one of which is the prescription of a second anti-psychotic medication in addition to clozapine. Still, new therapeutic approaches need to be developed [91].

The only drug with FDA approval for lowering the risk of repeated suicide conduct in individuals with schizophrenia or schizoaffective disorder is clozapine [38]. After prolonged use, it does not cause tardive dyskinesia, raise prolactin levels, nor cause any notable extrapyramidal adverse effects. Clozapine has several potentially fatal adverse effects despite its higher efficacy. One of those is the condition known as severe neutropenia, or agranulocytosis, which is characterized by an absolute neutrophil count less than 500/μL [92].

Xanomeline, a muscarinic receptor agonist, shows great potential in schizophrenia treatment. Xanomeline has a reduced but not insignificant affinity for the M2, M3, and M5 receptor subtypes and a partial selectivity for the M1 and M4 muscarinic receptors [6]. In animals treated with psychostimulant medicines that stimulate dopaminergic (DA) neurotransmission (e.g., amphetamine or apomorphine) or block N-methyl-d-aspartate (NMDA) receptors (e.g., phencyclidine [PCP], dizocilpine [MK-801], or ketamine), xanomeline demonstrates a robust “antipsychotic drug–like” effect. A number of preclinical behavioral models of “psychosis” and recent pharmacological Magnetic Resonance Imaging (MRI) studies have confirmed xanomeline’s putative antipsychotic properties, which include its capacity to reduce the functional effects of dopaminergic and glutamatergic psychostimulants [93]. In comparison to cell lines expressing M3 or M5 receptors, xanomeline exhibits higher efficiency and potency in M1 receptor-containing cell lines. On the other hand, xanomeline’s ability to block the forskolin-stimulated increase in cyclic adenosine monophosphate (cAMP) in an M2-expressing cell line is less strong and effective, highlighting its functional M1 selectivity [94].

The existence of M4 and/or M2 autoreceptors on cholinergic afferents may be the reason behind xanomeline’s suppression of the Ventral Tegmental Area (VTA) DA neuron firing rate while having no effect on the substantia nigra DA neuron firing rate. These results are significant because it seems that xanomeline selectively inhibits the midbrain dopaminergic (DA) circuits responsible for antipsychotic-like activity and not those responsible for controlling motor side effects [93].

An experimental medication called KarXT (xanomeline–trospium; Karuna Therapeutics TM, Boston, MA, USA) offers early promise in treating both positive and negative symptoms of schizophrenia. Trospium, a peripheral muscarinic receptor antagonist licensed by the US Food and Drug Administration that does not pass the blood–brain barrier, is combined with the M1/M4-preferring muscarinic receptor agonist xanomeline in KarXT. Due to its notably polarized tertiary amine structure, trospium is incapable of infiltrating the central nervous system. The union of xanomeline and trospium (Table 1), denoted as KarXT, displays promise in schizophrenia treatment, as trospium—a class of antimuscarinic agent—is expected to mitigate xanomeline’s cholinergic adverse effects. This combination exhibits potential in diminishing cholinergic side effects by a notable 50%, as observed in healthy volunteers. When compared to xanomeline alone, the co-administration exhibited an enhanced safety profile. Numerous preclinical studies indicate that M1 muscarinic receptor agonists can alter the neural circuits in the brain linked to memory, learning, and attention [95].

Because of its distinct mode of action, xanomeline may be helpful for about one-third of patients who did not respond to existing medications. Since it has demonstrated improvements in both positive and negative symptoms, xanomeline presents a new alternative to existing medications [39,96].

## 7. M4 Subtype Selectivity

**Figure 11:**
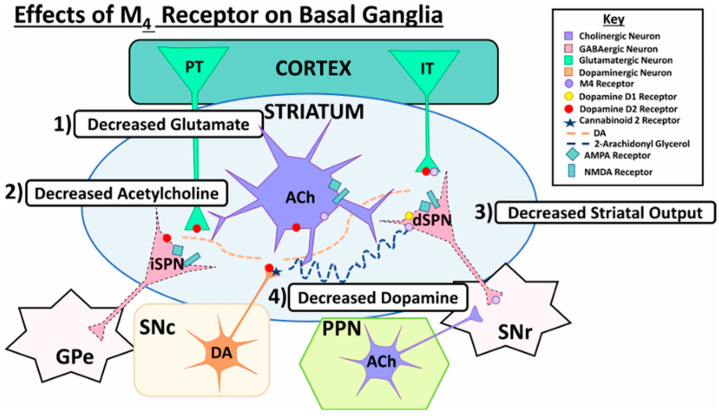
Routes for M4 movement modulation, from [98].

Recent progress in the understanding of functions attributed to each subtype of mAChR in the context of disease pathology proposes that the potential behind the antiparkinsonian and antidystonic effects observed using nonselective antimuscarinic treatments might lie in the development of highly specific ligands tailored to individual subtypes [45]. M4 receptors belong to the metabotropic acetylcholine receptor family, linked to Gαi/o proteins. Activation of M4 by acetylcholine results in adenylyl cyclase inhibition, causing a reduction in cAMP levels. M2 muscarinic receptors present high similarity in function to M4 ones. However, they are not an ideal pharmacological target for the treatment of central nervous system diseases because of their existence in peripheral heart and lung tissues, which can lead to safety issues [97]. According to multiple lines of evidence, M4 is the main mAChR subtype responsible for controlling dopamine-release signaling in the basal ganglia and associated motor activities (Figure 11) [45].

Firstly, M4 suppresses glutamate release at corticostriatal terminals. Secondly, it is believed that M4 prevents striatal cholinergic interneurons (ChIs) from releasing ACh. Thirdly, M4 directly inhibits Gαolf-coupled DA D1 receptor (D1) signaling and promotes long-term depression (LTD) in direct-route spiny-projection neurons (dSPNs) by acting on their cell bodies and terminals. The likelihood of DA release through autacoid signaling, specifically, the release of the endocannabinoid 2-arachidonoylglycerol (2-AG), which subsequently binds to Gαi/o-coupled CB2 cannabinoid receptors (CB2) on nigrostriatal terminals, is likewise decreased when ACh binds to M4 on dSPN cell bodies [98].

In diseases such as Parkinson’s disease and dystonia, the amount of dopamine release or signaling is noticed to be low; thus, non-selective mAChR antagonists are efficient in calming the motor dysfunctions. It is yet to be confirmed, but recent data indicate that mAChR subtype-selective medications, most likely M4, may preserve the therapeutic effectiveness of non-selective antagonists in movement disorders while preventing side effects [41]. This leads to a prolonged decrease in striatal dopamine release. Furthermore, intact signaling through CB2 cannabinoid receptors is necessary for both the long-term suppression of dopamine release brought on by M4 activation and the antipsychotic-like effects of M4 activators [99].

**Figure 12:**
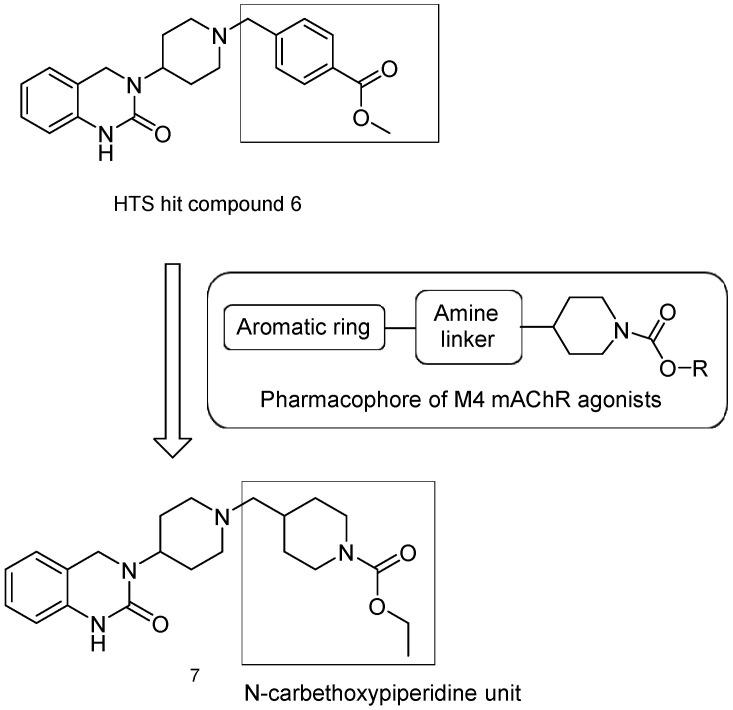
N-carbethoxypiperidine is considered as a functional group (pharmacophore group) for M4 mAChR activation. In compound 7, N-carbethoxypiperidine was the substitute for the benzyl group in compound 6, from [42].

There has been recent research in the development of dual M1/M4 agonist, by identifying scaffolds through high-throughput screening and combining them with known pharmacophores of M4 PAMs to reveal lead compounds. Their research was mainly focused on dihydroquinazolinon [42], 7-azaindoline [43], and N-substituted oxindole. These compounds were evaluated in vitro and showed promising characteristics. Although since then no further data have been reported on these compounds, the research has revealed the structural elements necessary for conferring M4 subtype specificity, such as a basic piperidine core with a terminal ethyl carbamate functional group (Figure 12, Table 1) [44].

In 2021, a study unveiled the identification, examination, and definition of novel selective M4 antagonists—namely, VU6013720, VU6021302, and VU6021625 (Table 1). The study also validated the enhanced effectiveness of these optimized compounds in alleviating symptoms of movement disorders, such as Parkinson’s disease and dystonia, as demonstrated in both pharmacological and genetic models. Among these compounds, VU6021625 stands out as the most potent and specific, boasting significantly enhanced pharmacological attributes compared to previously documented compounds. It demonstrates enhanced functional and binding selectivity, accompanied by minimal off-target activity at micromolar levels. Of particular significance, VU6021625 has demonstrated the capability to counteract the impacts of mAChR agonists on basal ganglia activity and dopamine release in mouse brain slices [45].

**Figure 13:**
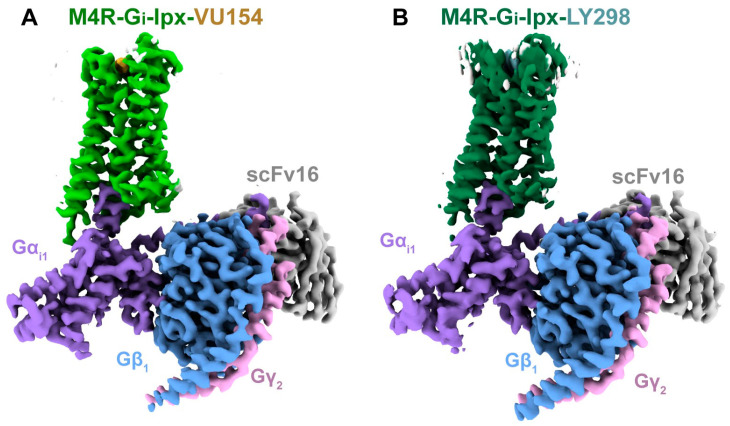
Cryo-electron microscopy (cryo-EM) structures for determining the 3D structure of biomacromolecules and complexes. (**A**). Cryo-EM map of VU154-Ipx. (**B**). Cryo-EM map of LY298-Ipx-, from [46].

**Figure 14:**
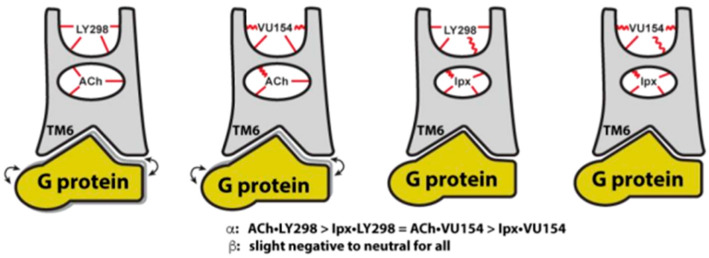
VU154 and LY298 had stronger binding affinity to M4R with Ach instead of Ipx. Furthermore, LY298 is a more potent allosteric positive modulator than VU154, from [46].

LY 2033298 (LY298) (Table 1) is a positive allosteric modulator of the M4 receptor, and it showed high efficacy in schizophrenia treatment in preclinical studies. Although it was a promising molecule, there was difficulty in optimizing the chemical structure of the compound while maintaining its binding ability to the receptor; thus, the increase in the therapeutic index was difficult to achieve. The continuous effort for chemical structure optimization led to the discovery of another positive allosteric modulator (PAM), VU0467154 (VU154) (Table 1). Key parameters were identified that characterize signaling and allostery for these ligands by analyzing the pharmacology of the PAMs LY298 and VU154 with the agonists ACh and Iperoxo (Ipx) across radioligand binding assays and two distinct signaling assays (Figure 13). Experiments indicated that VU154 and LY298 had a similar affinity for binding on the allosteric sites. Ipx was used to create more M4R-Gi1 complexes, with or without the co-addition of either LY298 or VU154, because of its greater affinity than Ach (Figure 14) [46].

In the PAM agonist receptor–transducer bound conformation, Gaussian accelerated Molecular Dynamics (GaMD) simulations showed that LY298 had a more stable binding pose and interactions with the receptor than VU154. There is a paradox between ligands, since they display more stable binding affinity to the receptor, but simultaneously lower efficiency [46].

### 7.1. M4 and M5 Muscarinic Receptors

It is known that between the M1 and M5 muscarinic receptors, there is high homology, thus leading to difficulty in seeking molecules that will bind to specific subtypes. In the need for new drugs, M4 and M5 muscarinic receptors were studied, as well as the mechanisms of targeting them. The two compounds that were investigated, AQ-RA741 and (S)-ML375 (Table 1), will be the base of future research in potential M4 or M5 selective antagonists.

**Figure 15:**
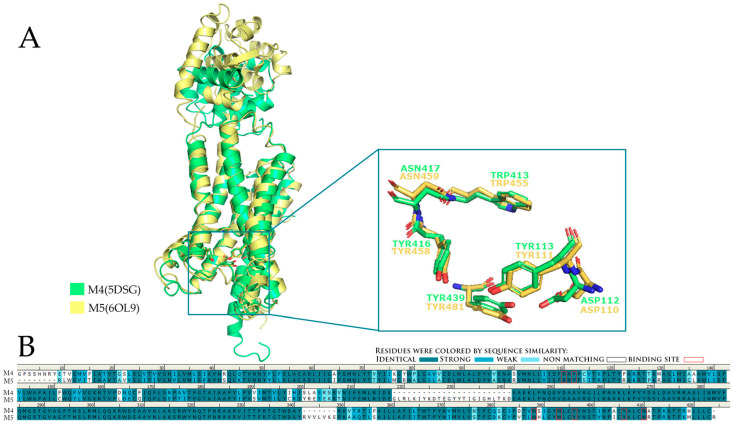
(**A**). The essential amino acid residues and 3D crystal structures of M4 and M5 receptors. (**B**). Sequence alignment of M4/5. Residues with dark-blue color are identical in M4 and M5 receptors, and amino acids shown as light-blue color are indicated as similar, from [47].

The insertion sites of M4 and M5 muscarinic receptors present a high grade of similarity. For instance, the amino acids ASP112M4, TYR113M4, TRP413M4, TYR416M4, ASN417M4, and TYR439M4 are relative to residues ASP110M5, TYR111M5, TRP455M5, TYR458M5, ASN459M5, and TYR481M5 (Figure 15). The similarity of residues order among M4 and M5 receptors is around 70.0%, and the general similarity of two receptors can arrive at 81.1% [25].

**Figure 16:**
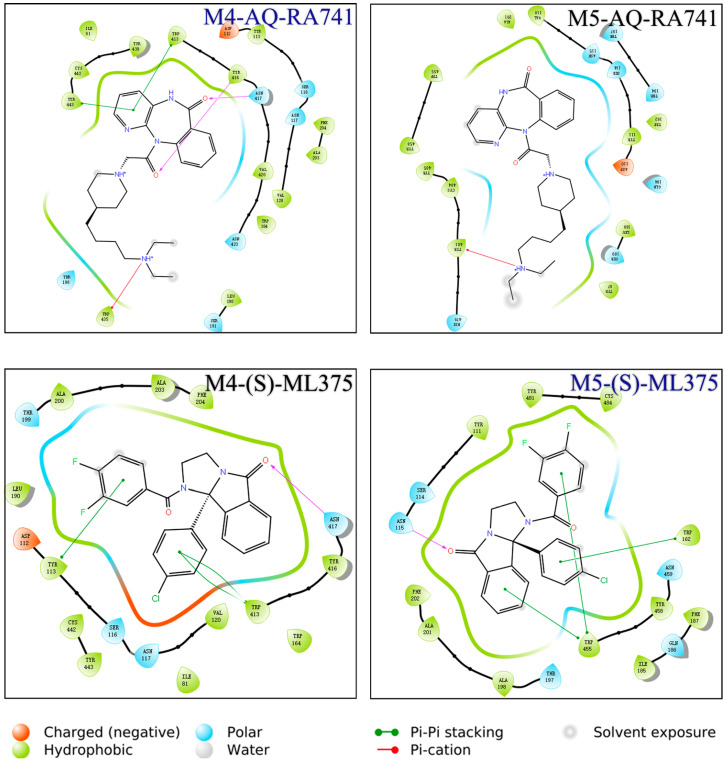
Interactions between potent selective antagonists and key amino acids of the active insertion sites, from [47].

Four binding groups are needed, including M4/AQ-RA741, M5/AQ-RA741, M4/(S)-ML375, and M5/(S)-ML375, so the antagonists will have the highest affinity as possible to the insertion sites of the receptors. Although the residues among M4 and M5 receptors are very similar, the interactions developed in cavities are quite different. Specifically, strong hydrogen bonds occurred between AQ-RA741 and amino acids TYR416M4 and ASN417M4, whereas there is no interaction with the respective residues TYR458M5 and ASN459M5. Furthermore, pi–cation interaction is formed between the nitrogen cation of AQ-RA741 and amino acid TRP435M4, but no interaction is noticed with TRP477M5. Three circles existing in the structure of AQ-RA741 interact with hydrophobic bonds with a hydrophobic loop of the M4 receptor, whereas they form no hydrophobic bond with amino acids TRP455M5 and TRP477M5. Generally, more bonding complexes are noticed between AQ-RA741 and the M4 receptor (Figure 16). Regarding the M5-selective antagonist (S)-ML375, hydrogen bonds are formed with the amino acids ASN115M5 and ASN417M4. In addition, pi–pi interactions are noticed between the benzenes of (S)-ML375 and TRP162M5 and TRP455M5, whereas the two benzene rings having halogens are substitutes, which interact with pi–pi bonds with TYR113M4 and TRP413M4. Hence, selective antagonism of M4/M5 by (S)-ML375 is enhanced by strong hydrogen bonds and pi–pi bonds (Figure 16) [25].

**Figure 17:**
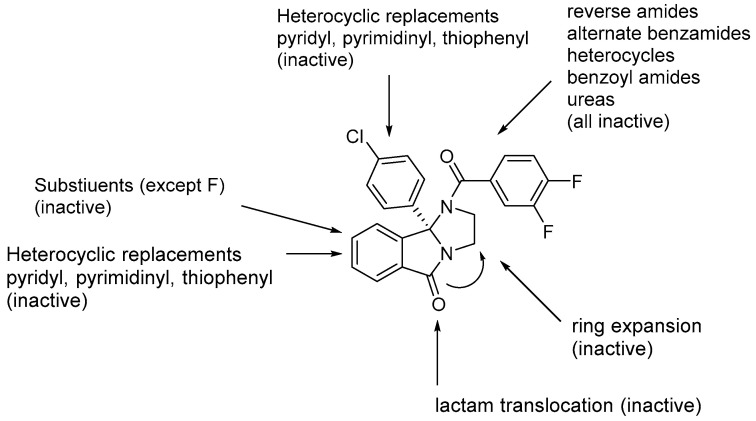
Efforts of SAR modulations of ML375, which led to ineffective analogs, from [48].

Although ML375 was undoubtedly a very important molecule for comprehending the interactions between the substance and the receptors, it had an extended elimination half-life; hence, there was a need for researching an M5 negative allosteric modulator with similar efficacy to ML375 but a lower elimination half-life (Figure 17) [48].

Research efforts were focused on modulation of the 9b-phenyl group, replacing the chlorine atom with amine (Table 1). This change could induce salt formation, optimizing DMPK characteristics, chirality ability, and the advantage of a great variety of amines [48].

### 7.2. Μ5 Muscarinic Receptor as Target for Treatment of Drug Abuse

Apart from the clear connection between M5 mAChR and schizophrenia, there has been interest in the importance of the M5 muscarinic receptor in drug dependence and addiction. The phenotypic analysis of Cholinergic Receptor Muscarinic 5 (Chrm5)-knockout (KO) mice indicated that the antagonism of the M5 receptor might be useful for cocaine and opioids use. Recent studies underlined the importance of signaling through M1 and M4 receptors for drug independence behavior [3].

Dopaminergic pathways are related to satisfaction and drug-induced reward. The striatum’s mAChRs regulate the dopamine released there. Experiments on K+-stimulated dopamine release from superfused striatal slices generated from Wild Type (WT) mice and M1 through M5 AChR KO mice have been carried out to demonstrate the impact of muscarinic receptor agonist oxotremorine. Results showed that M5 and M4 receptors play a vital role in the stimulation and release of dopamine, whereas M1 and M2 do not have an equally important role, and the M3 receptor has the opposite effect of inhibiting the dopamine release. To comprehend the physiological and neurological side of this muscarinic subtype, M5 receptor-deficient mice (M5 −/− mice) were generated and analyzed due to the unavailability of selective M5 receptor agonists [100]. It is found that in M5 −/− mice, morphine did not induce the rewarding and satisfying effects. In addition, naloxone-induced withdrawal symptoms were reduced physically as well as psychologically [101].

#### 7.2.1. Opioids

Both the “tail-flick” and the “hot-plate” tests were used to evaluate morphine’s analgesic qualities. These tests measure the latency to react to the noxious heated stimuli (a heated surface at 55 °C in the case of the hot-plate test, or a tail-focused beam from a halogen lamp in the tail-flick test). A similar degree of pain tolerance was induced by dose-dependent morphine in both WT and M5-deletion animals. Using the condition place preference method, which allows mice to enter both a drug-paired and a placebo-paired setting, the rewarding element of morphine usage was evaluated. Mice generally avoid the drug withdrawal environment, which makes them feel uncomfortable and aversive, and instead favor the drug-paired setting. According to the length of time spent in the morphine-associated chamber as opposed to the vehicle-associated chamber, the WT mice showed a dose-related (2.5–25 mg/kg i.p.) preference for the morphine-associated chamber. Nevertheless, in homozygous M5-deletion mice, morphine did not cause place preference at doses below 25 mg/kg [100].

Precipitated naloxone symptoms, such as leaping, wet-dog shakes, and teeth chattering, were seen in both WT and M5-deletion mice; however, in the latter group, the symptoms were less severe. The conclusive aspects are that a M5 AChR antagonist could lessen the drug-rewarding and withdrawal symptoms of morphine and other opioids, but would not affect the analgesic properties [100]. The molecule VU6019650 (Table 1) was recognized as a novel M5 orthosteric antagonist with efficacy, high selectivity for the M5 subtype receptor, and advantageous physicochemical characteristics in preclinical addiction models for systemic dosage [49].

#### 7.2.2. Cocaine

It is well known that the dopamine released from dopaminergic nerve terminals is what provides cocaine users with its satisfying effects. These neurons’ axons come from dopaminergic locations in the ventral tegmental region and substantia nigra. There are studies that connect the M5 AChR to cocaine or drug abuse. M5-deficient mice were used due to the unavailability of ligand to bind to M5 AChR as an agonist. It was noticed that the M5-deficient mice that had free access to cocaine had lower administration levels than the WT mice. Moreover, compared to WT mice, M5-deficient mice spent significantly less time in the drug-paired environment when given the option to remain in the vehicle- or cocaine-paired environment. Lastly, compared to WT mice, M5-deficient mice’s withdrawal symptoms were less unpleasant [100].

**Figure 18:**
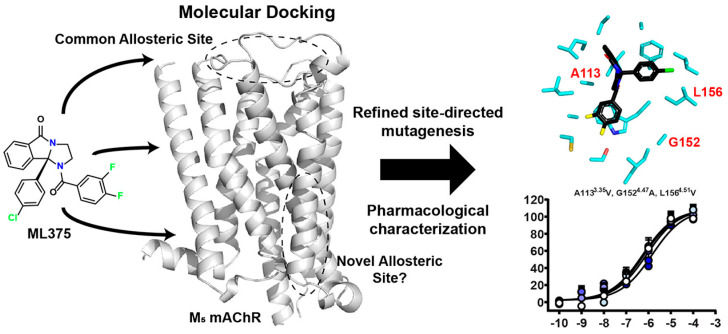
Molecular docking of molecule ML375 for M5 mAChR binding ability and pharmacological estimation, from [103]. The colors in the chart present the different M5-M2 mAChR chimeras used for prediction of potential allosteric sites within the transmembrane domain of M5 mAChR. This study highlights the ability of an allosteric modulator to target a different binding site of a highly conserved protein.

For the rat and human M5 mAChR, a selective negative allosteric modulator with improved pharmacokinetic characteristics for systemic dosage in rats has been discovered (ML375) [102]. The binding location of ML375 is still unclear, so a radioligand was employed to determine the binding site. This revealed that ML375 is not related to either the second allosteric site or the common allosteric site of M5 mAChR, which are both located in the extracellular vestibule of the receptor (Figure 18) [103]. The chemical structure of ML375 was modified, aiming at enhancement of CNS dispersion and DMPK properties (Table 1) [50].

The difficulty in synthesizing many M5 muscarinic antagonists is caused by the high similarity of residues among isoforms, thus making it difficult to target selectively one subtype without causing more adverse effects. Important chemical interactions include hydrogen bonding and pi–cation interactions, as well as the hydrophobic pocket formed by the essential residues—particularly CYS484 of M5—that have been revealed by silico investigations. Additionally, the water around ASN459M5 can be substituted with substituent groups, forming a network of hydrogen bond interactions through the simulation of bridging water. This replacement may not contribute to the increase in affinity with the allosteric site of the receptor, but it may lead to selective inhibition of M5 or even M4 receptors. Regarding the inhibition of M5 muscarinic receptor and taking into account the hydrophobic pocket, hydrophobic compounds may bind preferably to the hydrophobic pocket via hydrophobic interactions, thus providing a basis for future M5 selective antagonists [102].

A library of 98,000 chemicals was visually screened using shape-based scores as well as the quantitative structure–activity relationship (QSAR). Although neither strategy worked well by itself, a consensus score that combined the two techniques produced a novel scaffold. This scaffold, known as VU0549108 (Table 1), showed poor inhibitory action against the M5 mAChR and moderate selectivity. Furthermore, synthetic analogs from VU0549108 displayed low activity [51].

According to corresponding research, M1 muscarinic receptors are essential for both the analgesic effects of drugs and their behavioral side effects. Similarly, morphine has a stronger analgesic effect in M1-deficient mice, although the mice’s response to cocaine and morphine was lessened. Similar results were taken in parallel studies using the M1 antagonist pirenzepine [104].

## 8. Cancer

Acetylcholine (ACh) plays a vital role in various functions in the CNS, but recent studies indicate the importance of ACh in cancer. Apart from being a neurotransmitter, ACh seems to regulate cell proliferation, thus enhancing the tumor growth by autocrine and paracrine signaling [105]. Muscarinic subtypes are shown to have an impact in the development of gastrointestinal cancer [106]. MAChRs have a significant impact on the tumor microenvironment, affecting angiogenesis and immunological responses. Developing tailored therapeutics to stop mAChR-mediated carcinogenesis requires an understanding of these pathways. Significant regional diversity in mAChR density and functionality can be seen in the GI tract’s distribution. It is noteworthy that M2R and M3R are both significantly concentrated in the gut’s muscle layers, where they are essential for controlling intestinal motility, including peristalsis. Additionally, these receptors play a significant role in the enteric nervous system and alter neurotransmission, which in turn alters gut function in general [107].

**Figure 19:**
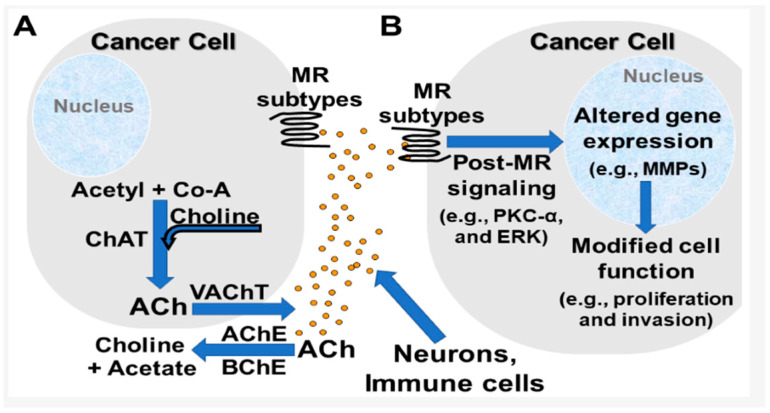
ACh released by cancel cells by membrane transporters (VAChT) functions as a ligand to many muscarinic receptor subtypes found in cancer cells. This agonism induces activation of many kinases and signaling pathways that modify the gene expression and enhance cancer cell proliferation, invasion, and metastasis. (**A**). The enzymes (choline acetyltransferase, ChAT) and transporters (vesicular acetylcholine transporter, VAChT) required for the production and release of ACh are expressed by neurons, immune cells, and cancer cells. In the extracellular space, acetylcholinesterase (AChE) and butyrylcholinesterase (BChE) quickly hydrolyze ACh to acetate and choline. (**B**). Muscarinic receptor (MR) subtypes that are expressed by nearby cancer cells are activated by ACh. Several protein kinases (like protein kinase C-α, PKC-α) and transcription factors (like extracellular signal-regulated protein kinase 1/2, ERK1/2) are activated by post-muscarinic receptor signaling, which changes the expression of genes encoding proteins that alter cell function and encourage cancer cell proliferation, survival, migration, invasion, and metastasis, from [108].

Specifically, M3 receptors seem to become overexpressed in gastric adenocarcinoma cells, and this overexpression is connected to metastasis through the lymphatic system. This can be prevented by darifenacin, which is an M3R antagonist. Respectively, absence of M3R obstructs cancer cell multiplication. Furthermore, even when ACh is not present to stimulate the M3 receptor, or through M3R antagonism, cancer cell proliferation is prevented. The production of nitric oxide is prevented by M3R antagonism in murine adenocarcinoma cells, while neoangiogenesis is suppressed by M1 and M2 receptor inhibitors. When M3R is activated, Epidermal Growth Factor Receptor (EGFR) is transactivated, and Mitogen-Activated Protein Kinase (MAPK)/Extracellular-Signal-Regulated kinase (ERK) signaling is activated downstream. Even though M3R antagonism tends to impede cancer growth, not the same results were revealed with M1R, M2R, and M4R antagonism. For instance, an M2 agonist called arecaidine inhibits the growth and migration of bladder cancer cells, and glioblastoma cell growth and lifespan are also suppressed in vitro by arecaidine propargyl ester [106]. Regarding pancreatic adenocarcinoma, M1R agonism indicated protection against tumor development, whereas M3R agonism promotes pancreatic cancer growth. Lastly, the development, invasion, and metastasis of colon cancer are all facilitated by M3R activation and post-receptor signaling. Under the EGFR/ERK and PKC/p38 MAPK pathways, M3R signal transduction induces and releases certain matrix metalloproteinases (MMP1, MMP7, and MMP10), which tear down the extracellular matrix to promote cell invasion (Figure 19) [108].

Oncogenic miRNAs, which have high basal expression levels already, are further elevated in colon cancer cells upon M3R activation. Thus, M3R activation and overexpression are responsible for dysregulation in miRNA expression [109].

Orthosteric muscarinic receptor agonists (M1R agonism) or antagonists (M3R antagonism) should be further investigated. The difficulty in aiming these receptors to the binding site with high selectivity and efficacy is caused by the high homology of residues in the binding location. Thus, molecules targeting non-conserved allosteric locations in the receptor could be novel therapeutic substances [108].

**Figure 20:**
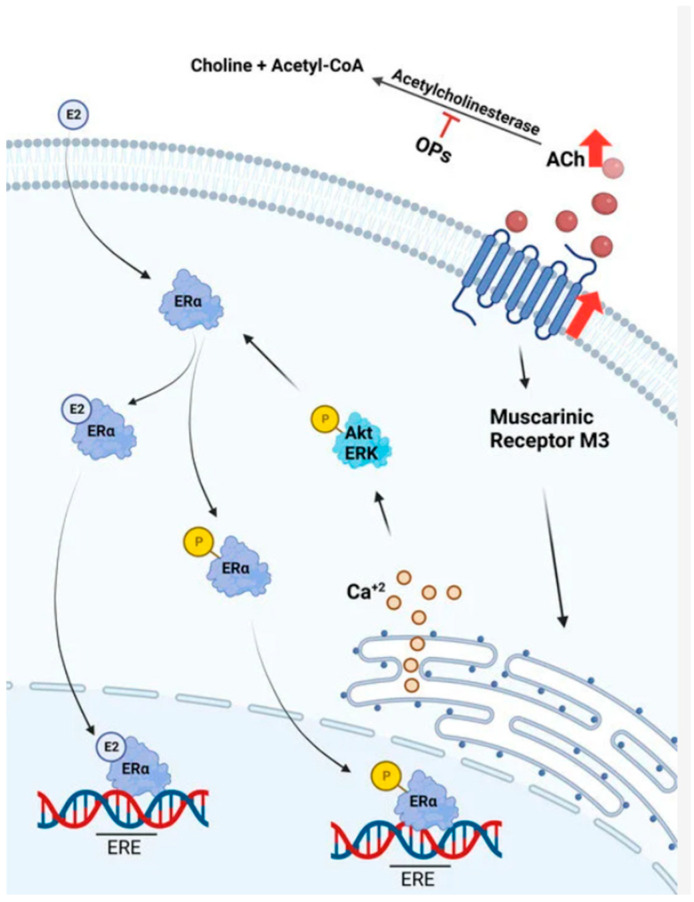
ACh activating M3 receptor, promoting ERa activity and cancer cell proliferation, from [105].

Increases in ACh availability can boost excessive expression of protein estrogen receptor alpha (ERa), whose role in breast cancer is vital (Figure 20) [105]. Breast cancer growth may be hindered by modulation of mAChR. Specifically, administration of paclitaxel combined with the muscarinic agonist carbachol in Michigan Cancer Foundation-7 (MCF-7) human breast adenocarcinoma cells showed dose-dependent apoptosis. Lidocaine and bupivacaine, muscarinic antagonists, were administrated intravenously in breast cancer surgery and caused caspases cleavage (especially caspases 7, 8, and 9), as well as poly ADP-ribose polymerase (PARP), leading to apoptosis of MCF-7 and MCF-10A cells [110].

Concentration of ACh promotes the release of Ca^+2^ and activation of the Phosphatidylinositol 3-Kinase (PI3K)/Protein Kinase B (Akt) and MAPK/ERK pathways [105]. The levels of nitrogen monoxide are higher due to activation of nitric oxide synthase. The proliferation of cancer cells is induced by nitrogen monoxide [106]. These alterations are linked to the recruitment of p-ERα to the nucleus and its induction. Nevertheless, ACh is unable to activate estrogen-responsive genes, which reveals that 17-β estradiol functions through a distinct mechanism. Lastly, ACh causes the upregulation of some Epithelial–Mesenchymal Transition (EMT) markers and increases the survivability of breast cancer cell lines [105].

The hypothesis about the connection between ACh and cancer had been suggested when organophosphates, such as parathion and malathion, caused mammary gland cancer in rats. High ACh concentration was linked to cancer growth. The agonism of ACh to muscarinic receptors causes activation of Gq/11 proteins and therefore an increase in the intracellular second messengers, DAG and 1,4,5-trisphosphate (IP3). PKC is activated by DAG, and intracellular Ca^+2^ is released into the cytosol by IP3. A multitude of signaling pathways, including the activation of PKC, Rapidly Accelerated Fibrosarcoma (Raf) kinase, and MAPK ERK1/2, are impacted by the cytosolic rise in Ca^+2^ [105].

Muscarinic receptors of cancer cells interact with IgG produced by breast adenocarcinoma cells having the action of an agonist, which contributes to cancer cell motility. Furthermore, nerve growth factor (NGF) regulation is controlled by acetylcholine, and the higher the NGF levels, the higher the possibility of cancer growth. Apart from the intense research for mAChR antagonists, inhibitors of choline acetyltransferase (ChAT) are vital for reduction in malignant tumor development, as well as cholinesterase activators. Acetylcholinesterase prevents fibroblasts from changing into myofibroblasts, which improves the viability and migration of cancer cells. Acetylcholinesterase activity is reduced in tumors, and this reduction is positively correlated with the tumor’s aggressiveness. However, two potent compounds as AChE inhibitors, eserine hemisulfate and bis 9-amino-1,2,3,4-tetrahydroacridine, had as a result the rapid multiplication of colon cancer cells [106].

Regarding the connection between muscarinic receptors and lung cancer, it is important to understand the difference in signaling between neuron and bronchial epithelial cells (BECs). The key difference is the role of choline high-affinity transporter (CHT1) in choline transport. Although CHT1 is expressed in BEC, CHT1 does not seem to be required for the transfer of choline in the production of ACh. In neurons, vesicular acetylcholine transporter (VAChT) and CHT1 play a role in packaging ACh into vesicles to get released. In BEC, VAChT and CHT1 do not contribute to the secretion, and their role is not known. Potential targets for treatment development in lung cancer include the stages involved in ACh production and signal transduction. Benefits are provided by muscarinic receptor antagonists and reduction of choline transport [14].

## 9. Conclusions

Undoubtedly, studying muscarinic receptors is a greatly interesting research field connected with the treatment of various diseases, for instance, Alzheimer’s disease, schizophrenia, cancer, and drug abuse. Each subtype has a significant role in signaling, and the agonism of some of them may worsen the state of disease. Thus, there is an urgent need to develop selective muscarinic receptor antagonists (or even agonists in some cases). Drug discovery may be enhanced by QSAR modeling and by AI applications, such as virtual screening, aiming at drug structure improvement and target recognition by identifying the potent lead compounds. To conclude, advancements in the pharmacological approaches involving muscarinic receptor agonists and antagonists hold significant potential for further enrichment, offering opportunities to refine their therapeutic applications, enhance selectivity, minimize side effects, and explore novel indications for treating a wide range of diseases and disorders. There is a huge unexplored field of study regarding connection of mAChR with several disorders. Ligand development and clinical trials conduction may highlight mAChR-based therapies as future therapeutic options.

## Figures and Tables

**Table 1:** Summary of muscarinic receptor agonists and antagonists that have been mentioned so far.

Target/Mechanism of Action	Reported Correlation with Disease	Compound	Structure	References
Inhibition of AChE, enhancement of Ach circulation through M1 receptor	AD	Tacrine	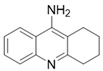	[25]
Inhibition of AChE; raise the hippocampal protein levels of PINK 1, NFASC, MYLK2, and NRAS	AD	Donepezil	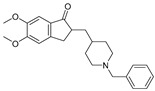	[25]
Allosteric activation of nAChR; activation of MARK, PI3K	AD	Galanthamine	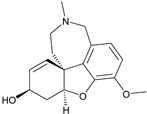	[25]
Inhibition of AChE and BChE	AD	Rivastigmine	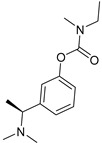	[25]
Allosteric agonism of M1 AChR	AD	AC-42	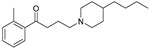	[30,31]
Agonism of postsynaptic mAChR within the cerebral cortex	AD	Arecoline	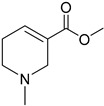	[31,32]
Direct agonism of mAChR	AD	Arecoline-based oxadiazoles	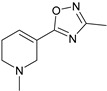	[32]
Direct agonism of mAChR	AD	Azanorbornane (1-azabicyclo[2.2.1]heptane)-methyl oxadiazole (exo)	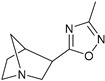	[32]
Direct agonism of mAChR	AD	Azanorbornane (1-azabicyclo[2.2.1]heptane)-methyl oxadiazole (endo)	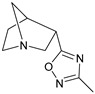	[32]
Direct agonism of mAChR	AD	Azanorbornane (1-azabicyclo[2.2.1]heptane)-amino oxadiazole (exo)	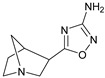	[32]
Direct agonism of mAChR	AD	Azanorbornane (1-azabicyclo[2.2.1]heptane)-amino oxadiazole (endo)	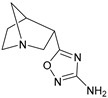	[32]
Orthosteric agonism of mAChR	AD	Alvameline	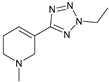	[32]
Orthosteric agonism of mAChR	AD	Milameline	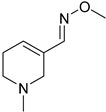	[32]
Orthosteric agonism of mAChR	AD	NGX-267	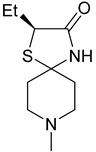	[32]
Orthosteric agonism of mAChR	AD	WAY-132983	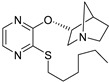	[32]
Selective agonism of M1 muscarinic receptor	AD	AF102B	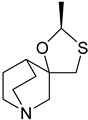	[33]
Selective agonism of M1 muscarinic receptor	AD	AF150(S)	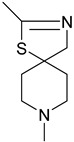	[33]
Selective agonism of M1 muscarinic receptor, activation of protein kinase C and ADAM17 via M1 mAChR	AD	AF267B	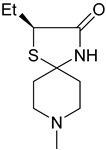	[33]
Orthosteric partial agonism of M1 mAChR	AD	HTL-9936	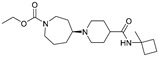	[32,34]
Positive allosteric modulation of M1 mAChR	AD	TAK-071	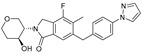	[35]
Positive allosteric modulation of M1 mAChR	AD	VU0486846	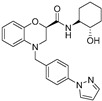	[36]
Partial agonism and antagonism of mAChR on different subunits of muscarinic receptor	Schizophrenia	Clozapine	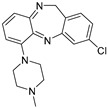	[37,38]
Small affinity for M2, M3, and M5 muscarinic receptors and partial selectivity for M1 and M4 receptors	Schizophrenia	Xanomeline	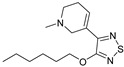	[6,39]
Peripheral muscarinic receptor antagonism	Schizophrenia	Trospium chloride	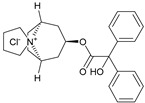	[39,40]
Activation of M4 mAChR	Parkinson’s disease and dystonia	N-carbethoxypiperidine	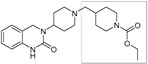	[41,42]
Increase in activation of M4 mAChR	Parkinson’s disease and dystonia	N-carbethoxypiperidine analogue with bulky substituents (basic piperidine core with a terminal ethyl carbamate functional group)	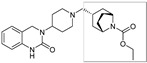	[43,44]
Increase in activation of M4 mAChR	Parkinson’s disease and dystonia	N-carbethoxypiperidine analogue with bulky substituents (basic piperidine core with a terminal ethyl carbamate functional group)	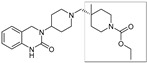	[43,44]
Selective antagonism of M4 mAChR	Parkinson’s disease and dystonia	VU6013720	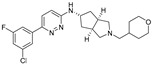	[45]
Selective antagonism of M4 mAChR	Parkinson’s disease and dystonia	VU6021302	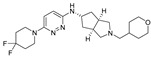	[45]
Selective antagonism of M4 mAChR	Parkinson’s disease and dystonia	VU6021625	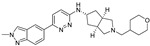	[45]
Positive allosteric modulation of M4 receptor	Schizophrenia	LY298	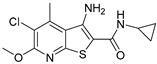	[46]
Positive allosteric modulation of M4 receptor	Schizophrenia	VU0467154	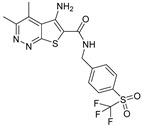	[46]
M4 or M5 selective antagonism	-	AQ-RA741	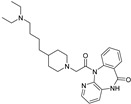	[47]
M4 or M5 selective antagonism	-	(S)-ML375	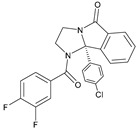	[47]
Negative allosteric modulation for M5 mAChR	-	Analogs of ML-375	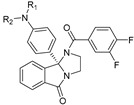	[48]
M5 orthosteric antagonism	Opioid abuse	VU6019650	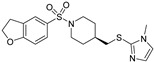	[49]
M4 or M5 selective antagonism	Drug abuse	Modification of ML375 structure—VU6000181	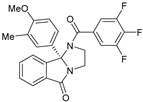	[50]
Poor inhibition of M5 mAChR	Drug abuse	VU0549108	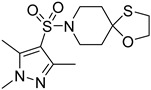	[51]
